# Special Analytical Health Commission on Tinned and Preserved Food

**Published:** 1906-06-30

**Authors:** 


### SPECIAL ANALYTICAL HEALTH COMMISSION ON TINNED AND PRESERVED FOOD.

### I. PRESERVED MEAT FOODS PREPARED IN ENGLAND.

We are pleased to be able to state that we have received a large number of samples from numerous leading makers of all kinds of preserved or partially preserved nitrogenous foods. As we pointed out in our article last week, it is especially in articles belonging to this dietetic class that the art of preservation is of such extreme use to the public. The Boer soldier fought for years upon biltong, which consists essentially of a dried and cured form of flesh meat. The German soldier in the Franco-Prussian war might equally truly be said to have fought on sausage. The present inquiry into the Army stores during the South African war has made us to some extent cognisant of the degree to which preserved meats are necessary in the commissariat of a fighting force, to say nothing of the question of emergency rations. Hence we make no excuse for choosing, as the first material for our investigation, preserved nitrogenous foods, or, in other words, foods which are intended to replace, or at any rate supplement, fresh meat; and in this connection it must be remembered that, although to the Londoner of to-day some of these articles are used to relieve the monotony of his diet or to stimulate a somewhat jaded appetite, we shall consider them not from this point at all, but simply as nutrients.

### Sausages.

The sausage is a very ancient and most praiseworthy article of diet. We think occasionally it is somewhat unnecessarily adorned, and regard the violent colour, which we are glad to say is generally confined to the skin, as rather detracting than adding to its appearance: all beauty is only skin deep, which is perhaps fortunate for the sausage, and in spite of its gaudy complexion we can say, speaking generally, that it contains sterling dietetic worth. In Germany sausages are legion, but in this country they are nothing like so varied in kind, and are generally made from raw meat suitably flavoured with various spices, and frequently incorporated with a small proportion of bread crumbs. The meat of fresh sausages usually contains some kind of harmless preservative which is really used instead of salt, and is generally a so-called boron preservative. It is not usual to dye the skins of fresh sausages, but it is very usual to submit the skins of smoked sausages to some dyeing agent, most often Bismarck brown or Congo red, and it is also quite usual even with fresh sausages to incorporate some colouring matter with their contents. We regard both these procedures, when kept within suitable bounds, as quite harmless. The public have their own idea as to what looks appetising, and have a perfect right to get any food, whether natural or preserved, the colour they like it. Practically all milk and all butter is artificially coloured, and the majority of us would immediately refuse a white butter or a white milk. Sausages differ largely in the amount of water and starchy matter they contain. The average composition of an English pork sausage is as follows:—Water, 51 per cent.; fat, 26.53 per cent.; nitrogenous substance, 11.42 per cent.; starch, 4.20 per cent.; ash, 3.84 per cent. For the sake of comparison we will also give here the average composition of raw beef, which is as follows: Water, 71.68 per cent.; nitrogenous matter, 20.52 per cent.; fat, 6.72 per cent.; ash, 1.21 per cent. From this we can gather that a sausage as an article of diet compares very favourably with an equivalent weight of meat. There is no waste in a sausage except the skin, and an average price for sausages may be taken as 9d. per lb., so it will readily be seen that the public gets good value for its money in the sausage. Perhaps the most economic nation in the world, from the dietetic standpoint, is the German, and the universality of the sausage in Germany ought to be a strong *a priori* recommendation for it to the British public.

We have received samples of fresh sausages from three well-known makers—namely, Messrs. Hudson Brothers, Limited, Oake Woods and Co., and Messrs. Harris. We have examined all these samples carefully, and have come to the conclusion that they are of good average composition; they do not contain an excess of water or starchy matter; the meat is of good quality, well minced, and we can detect no gristle or evidence that inferior parts of the carcase or extraneous matter has been introduced. All these sausages were of a pleasant flavour, and kept, both before and after cooking, a time quite adequate for ordinary retail consumption, even during the present hot weather. We think they can all be described as useful and wholesome articles of diet. Messrs. Hudson Brothers, Limited, have sent us their smoked sausages. These sausages are more concentrated than the fresh sausages, they have an excellent taste, and are thoroughly well made and wholesome. Messrs. Tebbutt and Co., the well-known Melton Mowbray makers, have forwarded a sample of their chicken, ham, and tongue sausage. From examination of this we have no reason whatever to doubt that it is made, as described, from the best chicken, ham, and tongue, and we regard it as an appetising and nutritious meat food.

All these makers have signified their willingness to have their factories inspected by our representative—a guarantee that their technique is above suspicion. Another variety of chicken, ham, and tongue sausage has been supplied by Messrs. Yallop and Co., Long Shore Works, Great Yarmouth. This sausage is contained in a cylindrical tin, and is so arranged that it can be gradually pushed up from the bottom for slicing purposes. We regard this product as in every way satisfactory.

### Soups, Beef-teas, Extracts and Essences of Meat.

In no class of foods has technology provided us with a greater variety than in bottled and tinned soups, beef-teas, extracts and essences of meat. Soups consist essentially of watery extracts of the respective meats. The object in making soup is to extract from the meat all those substances which are soluble in water at various temperatures.

### Soup-making.

The usual way of making soup is to place finely divided meat in cold water, allow it to stand, and then gradually to raise the temperature of the mixture, preventing evaporation. By this means the water extracts from the meat certain soluble meat basis and mineral matter. In, however, the making of most soup some chemical change of the nature of hydrolysis takes place in the meat, with the result that certain substances in the meat previously insoluble in water are rendered soluble, and thus the finished product comes to contain a varying quantity of gelatin, peptones, and albumoses. The actual amount of these substances, both absolutely and relatively, contained in the finished soup depends upon details of manufacture upon which we cannot enter here. One of the great arts of soup-making for invalids is to obtain a product free from fat; and one of the tests of a well made bottled or tinned soup is the absence of fat globules.

### Nutrient Value of Soups.

Concerning the nutritive value of soups, and for all practical purposes ordinary beef-tea may be included under this head, much has been written and said. When they simply contain mineral matters and meat extractives they must be more correctly regarded as stimulants than as foods. We do not for one moment, however, think that, even if they possess stimulant properties alone, they are the less valuable for doing so, at any rate in invalid dietetics with which we, as medical men, are most intimately concerned. In view, however, of giving the public maximum value for their money, manufacturers have not remained content with securing in their beef-teas or soups the presence of the soluble products of proteid hydrolysis—namely, albumoses, peptones, and gelatine, all of which certainly either supply energy to the body, or as in the case of gelatine are energy sparers—but have added to the finished product either divided meat as such or some of the meat fibre from which the soup or beef-tea was originally made.

### Beef-teas and Soups Examined.

We have received several samples of beef-teas and soups. Messrs. Lazenby have sent us a bottle of their beef-tea and a tin of their mock-turtle soup. The beef-tea is in the form of a clear jelly, and when gently heated with from $\frac{1}{3}$ to $\frac{1}{2}$ its volume of water, makes a clear, pleasant, and stimulating consommé, exceedingly well suited for patients whose digestive apparatus is in too irritable a condition to stand more digestive work. Messrs. Lazenby's mock-turtle soup is contained in a tin. We have examined it carefully; after the jelly is poured out the lining of the tin remains bright, and there is no reason whatever to suspect that any metallic contamination whatever has taken place. The same firm also supply an essence of beef in the form of a clear concentrated jelly. This essence is most suitable for invalids, and contains a high proportion of meat bases, which must rightly be considered the hall-mark of good beef essence or extract, just as volatile fatty acids must be regarded as the hall-mark of good butter.

We have been supplied with a unique, and, we think, exceedingly practical form of soup and *consommé* by Messrs. Cosenza and Co. These preparations are, we understand, made by Messrs. Maggi, the well-known makers of soup essences, which we have noticed before in these columns. The two preparations to which, however, we refer at the present time are *consommé* in tubes and tablets of soup. The *consommé* is in the form of solid cylindrical masses, each contained in a gelatine tube about an inch long and about $\frac{1}{8}$ of an inch in diameter, which is enclosed in a small cardboard box. Such a tube makes $\frac{3}{4}$ of a pint of excellent *consommé.* The tablets of soups are of various kinds, containing vegetables and rice. These desiccated tablets are treated differently according to the kind of soup they represent, but each makes approximately about $\frac{3}{4}$ of a pint, which we have tried and found to be of excellent quality.

Messrs. Tebbutt and Co., of Melton Mowbray, have sent us a tin of their finest quality kidney soup. We regard this in every way a sound product, carefully made and preserved.

From Messrs. Shippam, of Chichester, we have received a bottle of chicken broth jelly and specially prepared beef-tea. This jelly is essentially a chicken essence, and is to be taken in the solid form; it is quite clear, remains semi-solid at the present summer temperature, and has a pleasant taste. We have no reason to doubt that it is prepared in the most careful manner and from the best materials. We think the public may have every confidence in using it. This firm's beef-tea has every right to be called special, in that it contains not only those nutrient meat properties which are soluble in water, but also a considerable amount of solid nutritive nitrogenous matter. We shall have to consider some further samples of soups, meat, extracts, and meat essences in our next issue; it being our intention to report upon all home-manufactured products which may be forwarded to us for examination.

